# GPU-Enhanced DFTB Metadynamics for Efficiently Predicting Free Energies of Biochemical Systems

**DOI:** 10.3390/molecules28031277

**Published:** 2023-01-28

**Authors:** Anshuman Kumar, Pablo R. Arantes, Aakash Saha, Giulia Palermo, Bryan M. Wong

**Affiliations:** 1 Materials Science & Engineering Program, University of California-Riverside, Riverside, CA 92521, USA; 2 Department of Bioengineering, University of California-Riverside, Riverside, CA 92521, USA; 3 Department of Chemistry, University of California-Riverside, Riverside, CA 92521, USA; 4 Department of Physics & Astronomy, University of California-Riverside, Riverside, CA 92521, USA

**Keywords:** DFTB, metadynamics, GPUs, free energies, thermodynamics, cloud computing

## Abstract

Metadynamics calculations of large chemical systems with ab initio methods are computationally prohibitive due to the extensive sampling required to simulate the large degrees of freedom in these systems. To address this computational bottleneck, we utilized a GPU-enhanced density functional tight binding (DFTB) approach on a massively parallelized cloud computing platform to efficiently calculate the thermodynamics and metadynamics of biochemical systems. To first validate our approach, we calculated the free-energy surfaces of alanine dipeptide and showed that our GPU-enhanced DFTB calculations qualitatively agree with computationally-intensive hybrid DFT benchmarks, whereas classical force fields give significant errors. Most importantly, we show that our GPU-accelerated DFTB calculations are significantly faster than previous approaches by up to two orders of magnitude. To further extend our GPU-enhanced DFTB approach, we also carried out a 10 ns metadynamics simulation of remdesivir, which is prohibitively out of reach for routine DFT-based metadynamics calculations. We find that the free-energy surfaces of remdesivir obtained from DFTB and classical force fields differ significantly, where the latter overestimates the internal energy contribution of high free-energy states. Taken together, our benchmark tests, analyses, and extensions to large biochemical systems highlight the use of GPU-enhanced DFTB simulations for efficiently predicting the free-energy surfaces/thermodynamics of large biochemical systems.

## 1. Introduction

Molecular dynamics (MD) simulations are used to study a wide range of dynamic atomistic effects, including free energetics of chemical processes [1,2,3], protein folding [4,5], self-assembly [6], nucleation [7,8,9], glass formation [10,11], and chemical dynamics in solutions at interfaces [12]. The relevant physical processes in these studies are often rare events where a property of interest occurs on a time scale not accessible via simulation (within a reasonable amount of time) due to the presence of a large energy barrier separating local minima along the free-energy landscape. This well-recognized limitation of MD has led to the development of metadynamics approaches [13,14] to enhance the sampling of free-energy states and the rare events that allow the crossing of very high free-energy barriers [15]. Metadynamics is often applied in conjunction with classical molecular dynamics, where the atomistic interactions are approximated by classical force fields that are predetermined functions of the atomic coordinates.

When coupled with metadynamics, classical force field simulations of large systems can be used to estimate the structure and thermodynamics of relatively complex chemicals and materials. However, classical force fields can be inaccurate [3] and fail to capture the quantum interactions at the electronic level. For example, chemical reactions in which bonds are broken/formed cannot be directly simulated using the most common force fields [16,17]. Additionally, force fields are fitted to experimental data under specific conditions, which makes their transferability to other situations challenging [18,19]. To remedy these issues, ab initio metadynamics generated from density functional theory (DFT) calculations can be used to accurately capture bond breaking and formation in various chemical dynamics processes. In DFT-based metadynamics, interatomic forces are computed on the fly [20,21], leading to more computationally demanding calculations than classical metadynamics simulations. Moreover, the enormous computational cost associated with the DFT sampling of free energies restricts its applicability to relatively small chemical systems (less than 20 atoms) [22]. A promising alternative is the use of semiempirical methods such as density functional tight binding (DFTB), which can serve as a bridge between (efficient but inaccurate) MD and (costly but accurate) DFT calculations. In previous studies, our group and others have used DFTB calculations to gain computational speedups of up to 2–3 orders of magnitude compared with those of conventional DFT calculations [23,24,25,26,27,28,29,30,31].

In a previous work [28], we developed a massively parallelized heterogeneous CPU+GPU approach for carrying out large-scale DFTB MD simulations (2 ps) of an entire explicitly solvated protein (HIV protease) for the first time. Building on our experience with GPU-enhanced DFTB simulations of large biochemical systems, we now apply these techniques to long-term metadynamics simulations (10 ns). Because the computational bottleneck in metadynamics simulations is the diagonalization of the Hamiltonian matrix (which is performed several times during a single molecular dynamics trajectory) [28], many of our GPU-acceleration techniques can be harnessed for these calculations. To further accelerate our metadynamics calculations, we used massively parallelized cloud computing, which has recently emerged as a new computational platform for running large, complex electronic structure calculations. We first validate our approach by calculating the free-energy surface of alanine dipeptide (ADP), a chemical system typically used as a reference standard in the scientific literature for benchmarking metadynamics algorithms. Our GPU-based DFTB calculations are compared against the results obtained from classical force fields and hybrid DFT (PBE0) methods (the latter is the most accurate benchmark of ADP to date). Finally, to further extend our GPU-enhanced DFTB approach, we also carried out a 10 ns metadynamics simulation of remdesivir, which is prohibitively out of reach for routine DFT-based metadynamics calculations. Based on our benchmark tests, analyses, and extensions to large biochemical systems, we highlight the use of our GPU-based DFTB approach for accurately and efficiently predicting the free-energy surfaces/thermodynamics of large biochemical systems.

## 2. Theory and Methodology

### 2.1. DFTB Formalism

We briefly discuss the DFTB formalism in this section since it is used extensively to calculate free-energy surfaces/thermodynamics of biochemical systems in this study. Specifically, we used the third-order expansion of the Kohn–Sham (KS) DFT energy around a reference density, which is commonly referred to as DFTB3. To derive the DFTB3 total energy, the DFT total energy expression [32] is chosen as the starting point, which is given by [23]

(1) $$E\left[\rho \left(r\right)\right]=T\left[\rho \left(r\right)\right]+E_{\mathrm{ext}}+E_{H}+E_{\mathrm{nn}}+E_{\mathrm{xc}}\left[\rho \left(r\right)\right],$$

where *T* is the kinetic energy of the electrons, $E_{\mathrm{ext}}$ is the electron–nuclei interaction energy, $E_{H}$ is the mean-field (Hartree) energy, $E_{\mathrm{nn}}$ is the interaction energy of the nuclei, and $E_{\mathrm{xc}}$ is the exchange-correlation (XC) energy.

To obtain the expression of the DFTB3 total energy, a Taylor series expansion of Equation (1) around the reference density, $\rho^{0}\left(r\right)$, is carried out up to third order in the density fluctuations, δρ(r). The reference density is constructed as a superposition of atomic densities $\rho_{A}^{0}\left(r\right)$ on neutral atom A, i.e., $\rho^{0}\left(r\right)=\sum_{A}\rho_{A}^{0}\left(r\right)$. Substituting $\rho \left(r\right)=\rho^{0}\left(r\right)+\delta \rho \left(r\right)$ into Equation (1) and invoking a minimal basis set with a monopole expansion (among other approximations) [24], we obtain the DFTB3 total energy given as

(2) $$\begin{array}{cc} E_{\mathrm{DFTB}3} & =\sum_{i}^{\mathrm{occ}}\langle \psi_{i}|{\hat{H}}^{0}|\psi_{i}\rangle +\frac{1}{2}\sum_{\mathrm{AB}}^{M}\Delta q_{A}\Delta q_{B}\gamma_{\mathrm{AB}}^{h}+\frac{1}{3}\sum_{\mathrm{AB}}^{M}\Delta q_{A}^{2}\Delta q_{B}\Gamma_{\mathrm{AB}}+\frac{1}{2}\sum_{\mathrm{AB}}^{M}V_{\mathrm{rep}}^{\mathrm{AB}}\\ \; & =E_{\mathrm{BS}}+E_{\gamma }+E_{\Gamma }+E_{\mathrm{rep}}. \end{array}$$

The second, third, and fourth summations in Equation (2) run over the number of atoms, M, in the system. The first term, $E_{\mathrm{BS}}$, in Equation (2) is a sum over occupied orbital energies and corresponds to the band-structure energy. It can be obtained from the diagonalization of the non-self-consistent DFTB Hamiltonian ${\hat{H}}^{0}$, whose matrix elements are given by [33]:

(3) $$\begin{array}{c} H_{\mu \nu }^{0}=\left\{\begin{array}{cc} \epsilon_{\mu }^{\mathrm{free}\;\mathrm{atom}}, & \mathrm{if}\;\mu =\nu \\ \langle \phi_{\mu }|\hat{T}+\nu_{\mathrm{eff}}\left[\rho_{0}^{A}+\rho_{0}^{B}\right]|\phi_{\nu }\rangle , & \mathrm{if}\;\mu \in A,\;\nu \in B,\;A\neq B\\ 0, & \mathrm{if}\;A=B,\;\mu \neq \;\nu \end{array}\right. \end{array}$$

where $\phi_{\mu }$ and $\phi_{\nu }$ form a minimal Slater-type atomic basis, with μ and ν representing the indices of the valence atomic basis function associated with atoms A and B, respectively. In Equation (3), $\hat{T}$ is the kinetic energy operator, $\rho_{0}^{I}$ is the reference density of neutral atom I, and $\nu_{\mathrm{eff}}$ is an effective Kohn–Sham potential. To obtain $H_{\mu \nu }^{0}$, we first calculate $\phi_{\mu }$, $\phi_{\nu }$, and $\epsilon_{\mu }^{\mathrm{free}\;\mathrm{atom}}$ by solving a modified Kohn–Sham equation given by [34]:

(4) $$\left[-\frac{1}{2}{▽}^{2}+\;V^{\mathrm{eff}}\right]\phi_{\mu /\nu }\left(r\right)=\epsilon_{\mu /\nu }\phi_{\mu /\nu }\left(r\right),$$

where $V^{\mathrm{eff}}$ is the pseudoatomic potential, which includes the confinement potential [34].

Based on the form of Equation (3), only two-center elements are treated within the DFTB framework, which are explicitly calculated using analytical functions. Specifically, the Hamiltonian and overlap matrix elements are stored in Slater–Koster (SK) files for all pairs of chemical elements as a function of the distance between atomic pairs. As such, no explicit integral evaluation occurs during the simulation, which significantly improves the computational efficiency of the DFTB method [28,35]. The second term in Equation (2), $E_{\gamma }$, accounts for the charge fluctuation contributions to the energy, where $\gamma_{\mathrm{AB}}^{h}$ describes the effective on-site electron–electron interaction [34]. The third term, $E_{\Gamma }$, captures the changes in chemical hardness with respect to atomic charge, which improves the description of localized charges [36,37]. The last term, $E_{\mathrm{rep}}$, is a sum of pairwise repulsive functions, which are obtained by fitting to the DFT calculations of reference structures/molecules [38]. Similar to the Hamiltonian and overlap matrix elements, $E_{\mathrm{rep}}$ is pre-tabulated and stored in SK parameter files. By applying the variational principle, we obtain the Kohn–Sham equations [24]:

(5) $$\begin{array}{cc} \sum_{\nu }^{M}c_{\nu i}\left(H_{\mu \nu }-\epsilon_{i}S_{\mu \nu }\right)=0 & ,\;\nu \in B\;\mathrm{and}\;\forall A,\;\mu \in A,\;i \end{array}$$

(6) $$\begin{array}{cc} S_{\mu \nu }=\langle \phi_{\mu }|\phi_{\nu }\rangle & ,\;\forall \mu \in A,\;\nu \in B. \end{array}$$

The DFTB Hamiltonian, $H_{\mu \nu }$, in Equation (5) is given by:

(7) $$\begin{array}{c} H_{\mu \nu }=\langle \phi_{\mu }|{\hat{H}}_{0}|\phi_{\nu }\rangle \\ \;\;\;\;\;+S_{\mu \nu }\sum_{\xi }^{M}\Delta q_{\xi }\left(\frac{1}{2}\left(\gamma_{A\xi }+\gamma_{B\xi }\right)+\frac{1}{3}\left(\Delta q_{A}\Gamma_{A\xi }+\Delta q_{B}\Gamma_{B\xi }\right)+\frac{\Delta q_{\xi }}{6}\left(\Gamma_{\xi A}+\Gamma_{\xi B}\right)\right), \end{array}$$

where μ∈A, ν∈B, and $S_{\mu \nu }$ are the overlap matrix of the atomic orbitals; $\Delta q_{A/B}=q_{A/B}-q_{A/B}^{0}$ is the net charge of atom A/B. The summation in the second term of Equation (7) is performed over the number of atoms, M, in the system, and $\gamma_{\mathrm{AB}}$ is an analytical function of the interatomic distance. Because the atomic charges depend on the one-particle wave functions, $\phi_{i}$, Equation (5) must be iteratively solved by repeatedly diagonalizing the updated Hamiltonian until self-consistency is reached. This particular step is typically performed numerous times during a DFTB-MD simulation and accounts for 90–95% of the total wall time [39]. To overcome this computational bottleneck, we previously implemented a GPU-enabled eigensolver [28] to efficiently diagonalize the Hamiltonian in Equation (5), which is briefly described below.

### 2.2. Hamiltonian Diagonalization

As discussed in the previous section, the primary bottleneck in DFTB-based MD simulations is the diagonalization of the Hamiltonian matrix in Equation (7), which is typically performed numerous times along an MD trajectory [28]. The Hamiltonian diagonalization can be classified as a generalized symmetric definite eigenvalue problem of the form:

(8) A·x=λB·x,

where A and B are real and symmetric matrices, respectively; B is positive definitive; λ is the eigenvalue; and x is the eigenvector. Applying a Cholesky factorization on matrix **B** ($B=L\cdot L^{T}$, where **L** is a lower triangular matrix), Equation (8) can easily be reduced to a standard symmetric eigenvalue problem (C·y=λy, where $C=L^{-1}AL^{-T}$ and $y=L^{T}x$), which facilitates Hamiltonian diagonalization. Standard diagonalization routines can then be employed to solve the standard symmetric eigenvalue problem to obtain the eigenvalues and eigenvectors. In our previous study, we implemented GPU enhancements for the QR, Divide-And-Conquer, and RelativelyRoubust diagonalization routines [40] in an older version of the DFTB+ code [28]. In the DFTB v19.1 code [39], only the Divide-And-Conquer eigensolver routine is enhanced with GPU parallelization via the MAGMA library [41]. Since this particular routine is extensively used during the metadynamics simulations in our study (via Hamiltonian diagonalization, which occurs numerous times in each MD trajectory), we briefly review this routine in the following section.

### 2.3. Divide-and-Conquer

The Divide-And-Conquer eigensolver is based on recursively breaking down a problem into two or more sub-problems, which are subsequently solved to obtain a solution to the original problem [28]. This algorithm takes advantage of deflation [42], which occurs when an eigenpair of a submatrix of a tridiagonal matrix is an eigenpair of a larger matrix. After Equation (8) is reduced to a standard symmetric eigenvalue problem of the form C·y=λy, the matrix C is reduced to a block-tridiagonal matrix, **T**:

(9) $$T=\left(\begin{array}{cccccc} \; & \; & & 0 & 0 & 0\\ \; & T_{1} & \; & 0 & 0 & 0\\ \; & \; & & \beta & 0 & 0\\ 0 & 0 & \beta & \; & \; & \\ 0 & 0 & 0 & \; & T_{2} & \\ 0 & 0 & 0 & \; & \end{array}\right).$$

The Divide-And-Conquer approach uses the fact that a block-tridiagonal matrix is very close to a block-diagonal matrix [42], $\tilde{T}$, having the following form:

(10) $$\tilde{T}=\left(\begin{array}{cccccc} \; & \; & & 0 & 0 & 0\\ \; & {\tilde{T}}_{1} & \; & 0 & 0 & 0\\ \; & \; & & 0 & 0 & 0\\ 0 & 0 & 0 & \; & \; & \\ 0 & 0 & 0 & \; & {\tilde{T}}_{2} & \\ 0 & 0 & 0 & \; & \end{array}\right).$$

Because of its block-diagonal form, the eigenvalues and eigenvectors of the full matrix $\tilde{T}$ can be obtained from diagonalizing ${\tilde{T}}_{1}$ and ${\tilde{T}}_{2}$; as such, solving these two smaller problems is almost always faster than solving the original problem. First, the block-tridiagonal matrix, T, is written as a block diagonal matrix, $\tilde{T}$, plus a correction, C, i.e.,

(11) $$T=\;\tilde{T}\;+\;C$$

(12) $$T=\left(\begin{array}{cccccc} \; & \; & & 0 & 0 & 0\\ \; & {\tilde{T}}_{1} & \; & 0 & 0 & 0\\ \; & \; & & 0 & 0 & 0\\ 0 & 0 & 0 & \; & \; & \\ 0 & 0 & 0 & \; & {\tilde{T}}_{2} & \\ 0 & 0 & 0 & \; & \end{array}\right)\;+\;\left(\begin{array}{cccccc} 0 & 0 & 0 & 0 & 0 & 0\\ 0 & 0 & 0 & 0 & 0 & 0\\ 0 & 0 & \beta & \beta & 0 & 0\\ 0 & 0 & \beta & \beta & 0 & 0\\ 0 & 0 & 0 & 0 & 0 & 0\\ 0 & 0 & 0 & 0 & 0 & 0 \end{array}\right).$$

The eigenvalues and eigenvectors of ${\tilde{T}}_{1}$ and ${\tilde{T}}_{2}$ are subsequently calculated by recursively calling the Divide-And-Conquer algorithm. In the last step, the eigenvalues and eigenvectors of the original matrix T are built.

The Divide-And-Conquer algorithm scales as $O(n^{3})$ [43], where *n* is the matrix dimension. The steps used in the Divide-And-Conquer eigensolver are summarized in the following algorithm (Algorithm 1) flowchart [42].
**Algorithm 1:** The tridiagonal Divide-And-Conquer algorithm.
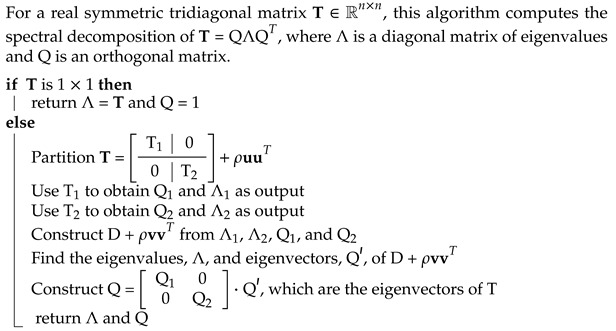


### 2.4. Metadynamics

Metadynamics is an accelerated sampling method that can be used to explore the free-energy landscape of a system as a function of collective variables (CVs) [13]. Within this formalism, a history-dependent bias potential composed of Gaussian functions is added to the Hamiltonian of the system. These external potentials “fill” the underlying free-energy basins, thus enabling an efficient exploration of the free-energy landscape. In well-tempered metadynamics (WT-MetaD) simulations, the Gaussian height is decreased during the simulation, which avoids overfilling the free-energy basins and ensures convergence of the final bias potential to the actual free energy (within a constant) [14,44]. As such, WT-MetaD simulations address the convergence problems associated with conventional metadynamics and allow the exploration of physically relevant regions of conformational space [45]. The WT-MetaD bias potential $V_{B}\left(s,t\right)$ at time *t* is constructed from the sum of Gaussian “hills” [14]:

(13) $$V_{B}\left(s,t\right)=\sum_{t^{\prime }=\tau ,2\tau ,\ldots }^{t^{\prime }<t}W\exp\left[\frac{-\beta V_{B}\left(s,t^{\prime }\right)}{\gamma }\right]\exp\left[-\sum_{i}\frac{{\left(s_{i}-s_{i}\left(t^{\prime }\right)\right)}^{2}}{2\sigma_{i}^{2}}\right],$$

where *W* is the initial height of the bias potential, τ is the time between deposited Gaussians, $\beta ={\left(k_{B}T\right)}^{-1}$ (where $k_{B}$ and *T* are the Boltzmann constant and temperature, respectively), γ is the bias factor, and $\sigma_{i}$ is the width of the Gaussians for the *i*th CV in the set, **s**, of collective variables. The first exponential term in Equation (13) decreases the height of the deposited Gaussians where previous bias potentials had been added. This reduction in the Gaussian height reduces the error and avoids exploring high free-energy states that are thermodynamically irrelevant [46]. The bias factor γ determines the rate at which the magnitude of the newly added potential decreases; a lower bias factor leads to a faster decrease in the bias potential. The last exponential is a product of Gaussians in the direction of the $i^{\mathrm{th}}$ CV with width $\sigma_{i}$ and centered at the CV value at time $t^{\prime }$. Using this approach, the system’s dynamics are enhanced, and different conformations are explored by adding an extra force (potential) to the system. The additional bias force for the $i^{\mathrm{th}}$ atom is given by [46]:

(14) $${\left.F_{i}^{B}\left(t\right)=\frac{\partial V_{B}\left(s,t\right)}{\partial s}\right|}_{s=s(t)}{\left.\frac{\partial s\left(r\right)}{\partial r_{i}}\right|}_{r=r(t)}.$$

In Equation (14), $V_{B}$ is the bias potential, **s** is a set of collective variables, r contains the position vector of all atoms, and $r_{i}$ is the position vector of the $i^{\mathrm{th}}$ atom. If the conformational space is sampled for a sufficiently long simulation time, the free-energy landscape over CV(F(s)) is obtained from the bias potential using the following expression [14]:

(15) $$\lim_{t\to \infty }V_{B}\left(s,t\right)=-\frac{\left(\gamma -1\right)}{\gamma }F\left(s\right).$$

## 3. Computational Details

The free energy, potential energy, and entropy landscapes of ADP and remdesivir were calculated using the GROMACS [47] and DFTB+ [39] software programs for classical and quantum metadynamics simulations, respectively. Both the classical and DFTB calculations were performed on a single remdesivir molecule without any explicit or implicit solvent. The following sections provide the detailed settings and parameters used in our study for each of these approaches.

### 3.1. Amber Calculations

All-atom molecular dynamics simulations for the remdesivir molecule were performed with the Amber ff19SB force field [48] and generalized Amber force field (GAFF) [49] parameters via AntechAmber to collect an overall ensemble with a 2 µs sampling. It is important to note that although classical force fields are parameterized and validated under explicit-solvent conditions, they are routinely used in calculations performed in vacuo [50,51,52]. Charge parameters for remdesivir were assigned using a restrained electrostatic potential (RESP) [53] charge in vacuo. The structure of remdesivir was obtained from the Protein Data Bank (PDB ID: 7BV2). The remdesivir structure was first optimized at the DFT/B3LYP/6-31G(d,p) level of theory using Gaussian 09 [54], and the RESP charges were calculated. All bond lengths involving hydrogen atoms were constrained using the SHAKE algorithm. Temperature control (300 K) was performed via Langevin dynamics [55] with a collision frequency of γ = 1 ps. The system was then subjected to energy minimization. The system was further heated from 0 to 100 K in a canonical ensemble (NVT) by running two simulations of 5 ps each and imposing position restraints of 100 kJ mol${}^{-1}$ Å${}^{-2}$. The temperature was further increased to 200 K in ≈100 ps of MD simulations in the NVT ensemble while reducing the restraint to 25 kJ mol${}^{-1}$ Å${}^{-2}$. Subsequently, all restraints were released, and the temperature of the system was raised to 300 K in a single NVT simulation of 500 ps. After ≈1.1 ns of equilibration, ≈10 ns of NVT runs were carried out. All classical MD simulations were performed with the GPU-enhanced version of AMBER 20 [56]. The well-equilibrated system was used as starting point for the subsequent well-tempered (WT) metadynamics [14] simulations.

A structural assessment of remdesivir was performed using metadynamics simulations, which determined the conformational preferences of the dihedral angles in the main scaffold. Gaussian hills with an initial height of 1.2 kJ mol${}^{-1}$ and a hill width of 0.35 kJ mol${}^{-1}$ were applied to the system. In this WT scheme, Gaussian functions were rescaled with a bias factor of 10. The temperature was kept constant by a V-rescale thermostat (NVT step) with a coupling constant of τ = 0.1 ps. The Lincs [57,58] method was applied to constrain covalent bond lengths, allowing an integration step of 2 fs. The GROMACS 2019.6 [47] software package interfaced with the PLUMED plugin package 2.6.4 [59] was employed, and the “sum hills” tool from the PLUMED package was used to compute the free-energy surfaces.

### 3.2. DFTB Calculations

All DFTB calculations in this study utilized high-performance computing hardware (40 Intel Xeon Platinum 8168 CPUs and 8 NVIDIA Volta V100 GPUs) executed on virtual machines (VMs) from Microsoft Azure cloud computing resources. Using the high-performance computing container maker (HPCCM) [60], an open-source tool for deploying the HPC components into container images, we created a docker image on the Azure cloud for DFTB v19.1 with the required libraries and dependencies (Intel MKL, Open MPI, Cuda, PLUMED v2.6 [59], and MAGMA v2.5.3 [41]). As such, this study also demonstrates the viability and readiness of cloud computing for high-performance computing workloads for first-principles computational approaches [61,62]. In the present study, we used the self-consistent-charge formulation of DFTB (SCC-DFTB) in its third-order scheme (DFTB3), which includes the third-order term in the DFT energy expansion around the reference density [36]. We used the 3ob-3-1 Slater–Koster parameter set and its corresponding Hubbard derivative parameters, which have been previously shown to work well for biochemical systems [63,64,65]. We included DFT-D3 dispersion effects [66,67] to accurately describe the London dispersion interactions in these biochemical systems. All the initial geometries for our metadynamics calculations were relaxed with nonperiodic boundary conditions (i.e., a cluster geometry), such that all the forces were less than 0.04 eV Å${}^{-1}$. All the DFTB calculations were performed without any implicit or explicit solvent. All subsequent metadynamics calculations were performed after running an NVT equilibration for 2 ps. For all the metadynamics runs, the temperature in the NVT ensemble (T = 300 k) was controlled using a Nose–Hoover thermostat [68,69]. Metadynamics calculations were performed with the PLUMED code [59] patched with DFTB+ [39]. All of our DFTB-based MD simulations used a time step of 1.0 fs, and all metadynamics calculations were carried out until the free energy converged with respect to each CV. In our metadynamics calculations, the height and width of the Gaussian hills were set to 1.2 and 0.35 kJ mol${}^{-1}$, respectively. The deposition rate of the Gaussian hills was 500 MD steps, and a bias factor of 10 was used. Finally, we used the “sum hills” tool in the PLUMED package to compute the free-energy surfaces. To obtain the potential energy/entropy surfaces, we calculated the local average of the internal energy computed on a (ϕ, ψ) grid using our in-house pandas-based [70] python scripts. To smoothen the noise in our energy/entropy surfaces for visualization purposes, we used a Gaussian filter.

## 4. Results and Discussion

### 4.1. Timing Benchmarks

To evaluate the computational speedup gained from our heterogeneous CPU+GPU DFTB metadynamics calculations, we benchmarked the timings for carrying out eight SCC iterations of the first MD step on protease (PDB ID: 6LU7), which consists of 5029 atoms. Table 1 compares the performance of various combinations of CPUs (Intel Xeon Platinum 8168) and GPUs (NVIDIA Volta V100) for performing eight SCC steps using the Divide-And-Conquer eigensolver in DFTB+. As shown in Table 1, the introduction of GPUs provides a significant speedup of nearly 140%. While this speedup is only for eight SCC steps in a single MD step, this improvement scales exponentially for full MD calculations because multiple SCC steps are performed during each MD step. It is also worth mentioning that increasing the number of GPU cores from two to four did not enhance performance. One possible reason is the steep communication overhead associated with data transfer from the CPU to GPU, which adversely affected computational performance. Moreover, the matrix dimension is “only” 12,642 × 12,642, which is too small for effective multi-GPU scaling. It is also interesting to note that increasing the number of CPUs from four to eight did not increase performance. One of the reasons for this is Amdahl’s law [71], which limits the scaling based on the dimension of the matrices being solved (which, in turn, depends on the size of the molecular system studied). Based on our benchmark timings (Table 1), a hardware configuration of two CPUs and two GPUs gave the best timings for calculations on protease 6LU7. We also performed similar benchmarks with both ADP and remdesivir and found that two CPUs and two GPUs also provided the most optimal hardware configuration for efficient metadynamics simulations.

### 4.2. Metadynamics Benchmarks on Alanine Dipeptide

**Figure 1:**
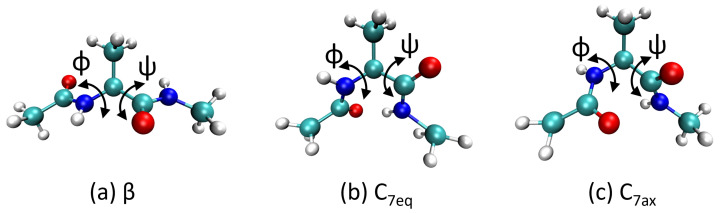
Molecular structures of three metastable minima: (**a**) β, (**b**) C${}_{7\mathrm{eq}}$, and (**c**) C${}_{7ax}$ of alanine dipeptide, which is composed of 22 atoms. Each panel depicts the two dihedral angles (ϕ, ψ) used to bias and analyze our calculations. The H, C, N, and O atoms are shown in white, cyan, blue, and red, respectively.

To assess the efficiency and accuracy of our DFTB-based metadynamics calculations, we first performed a benchmark analysis with alanine dipeptide (ADP). In the scientific literature, ADP is frequently used as the archetypal system to evaluate the performance of various enhanced sampling methods, including, for example, the extended harmonic superposition approach [72], replica exchange solute tempering [73], string methods [74], as well as numerous metadynamics approaches [14,75,76]. The most accurate first-principles calculation of the free-energy surface of ADP to date is an ab initio molecular dynamics (AIMD) simulation at the PBE0 level of theory by de Pablo et al. using the adaptive biasing force method [22]. Two dihedral angles, ϕ and ψ, shown in Figure 1, are chosen as the collective variables (CVs) and are used to describe the thermodynamics of ADP. Using these two dihedral angles as CVs, we were able to identify three different minima in a Ramachandran plot, which describes the peptide’s secondary structure. The first minimum, denoted as β, is located at (ϕ,ψ)=(−2.5,−2.5) radians and shows an almost-planar geometry. The second and third minima (located at (ϕ,ψ)=(−1.5,1.2) and (1.0,−1.2) radians, respectively) are stabilized by an intramolecular hydrogen bond and are denoted as C${}_{7\mathrm{eq}}$ and C${}_{7ax}$, respectively.

**Figure 2:**
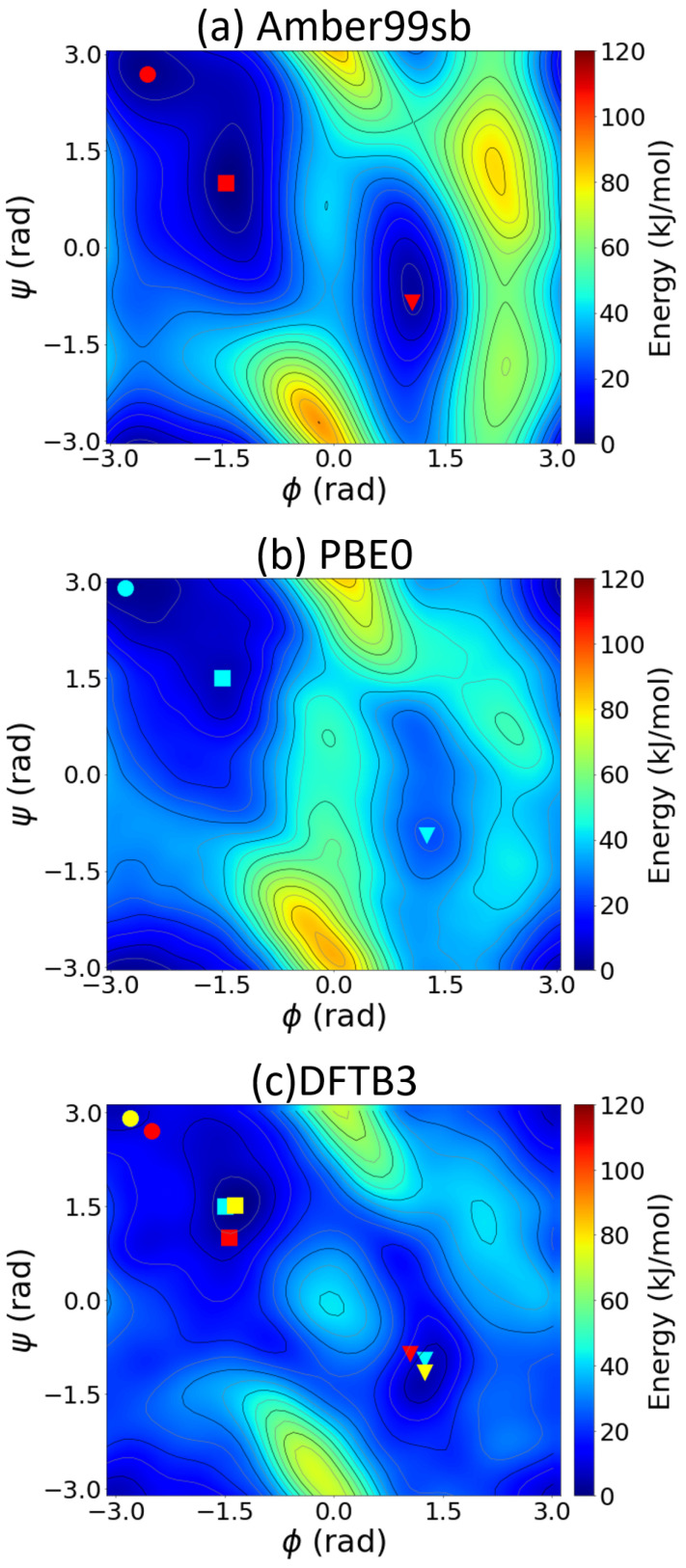
Two-dimensional free-energy surface of alanine dipeptide as a function of the backbone dihedral angles, ϕ and ψ, obtained from well-tempered metadynamics simulations using (**a**) classical MD with the Amber99sb force field, (**b**) DFT-PBE0 calculations, and (**c**) SCC-DFTB3 calculations. The red, cyan, and yellow points in panels (**a**–**c**) represent the local minima obtained using the Amber99sb force field, PBE0, and SCC-DFTB3, respectively. ●, ■, and ▼ denote the β, C${}_{7eq}$, and C${}_{7ax}$ minima/metastable structures, respectively.

Figure 2 depicts the FES of the alanine dipeptide projected onto the ϕ and ψ dihedral angles obtained from well-tempered metadynamics simulations at 300 K. Figure 2 compares the FES of ADP obtained with the Amber99sb classical force field (Figure 2a), DFT at the PBE0 level of theory (Figure 2b), and DFTB3 (Figure 2c). The data used to plot Figure 2a,b were taken from Ref. [22]. As described in Ref. [22], there are clear differences between the DFT-PBE0 and classical force fields. These differences in the FES are distinctly visible near the maximum located at (ϕ,ψ)=(2.3,1.2) radians in the Ramachandran plot, which is more pronounced in the AIMD calculations. In addition, the Amber99sb force field predicts a significantly larger barrier that spans the entirety of ψ at ϕ=2.2 radians, which restricts conformational transitions across the dihedral angle.

In contrast with the classical force field results, the FESs obtained with DFT-PBE0 and DFTB3 (Figure 2) have the same general morphology and show some similar trends. In particular, both DFT-PBE0 and DFTB3 predict the same local minima regions on the Ramachandran plots (the β, C${}_{7\mathrm{eq}}$, and C${}_{7ax}$ local minima/metastable states are indicated by the ●, ■, and ▼ markers, respectively, in Figure 2). Moreover, DFT-PBE0 and DFTB3 predict the same maxima regions on the FES, which are located at approximately ϕ=0 radians. Although morphologically similar, the DFT-PBE0 and DFTB3 FESs do exhibit some differences. DFTB3 predicts a much smaller barrier that spans the entirety of ψ at ϕ=2 radians, likely allowing conformational transitions across the dihedral angle. The most noticeable discrepancies between the DFTB3 and DFT-PBE0 FESs appear at the center of the plots at (ϕ=0, ψ=0) radians. The DFTB3 plot shows a maximum near (0, 0) radians that is surrounded by two valleys constituting natural pathways between the C${}_{7\mathrm{eq}}$ and C${}_{7ax}$ minima. The FES region near (0, 0) radians predicted by DFT-PBE0 shows no distinct local maxima; nevertheless, both DFTB3 and DFT provide similar geometries for the two C${}_{7ax}$ and C${}_{7\mathrm{eq}}$ conformations. Moreover, our energies and geometries are consistent with those in Ref. [77], where the authors also used DFTB to calculate the FES of ADP. Taken together, our results demonstrate that DFTB3 reproduces the main features of the DFT-PBE0 FES (despite slight underestimation of barrier heights); most importantly, finite-temperature configurational properties/energetics predicted by DFTB3 are typically more accurate than those predicted by classical force fields.

To understand the origin of the FES differences, we further investigated the contribution of internal energy and entropy to the free energy in the MD simulations. The change in free energy is given by

**Figure 3:**
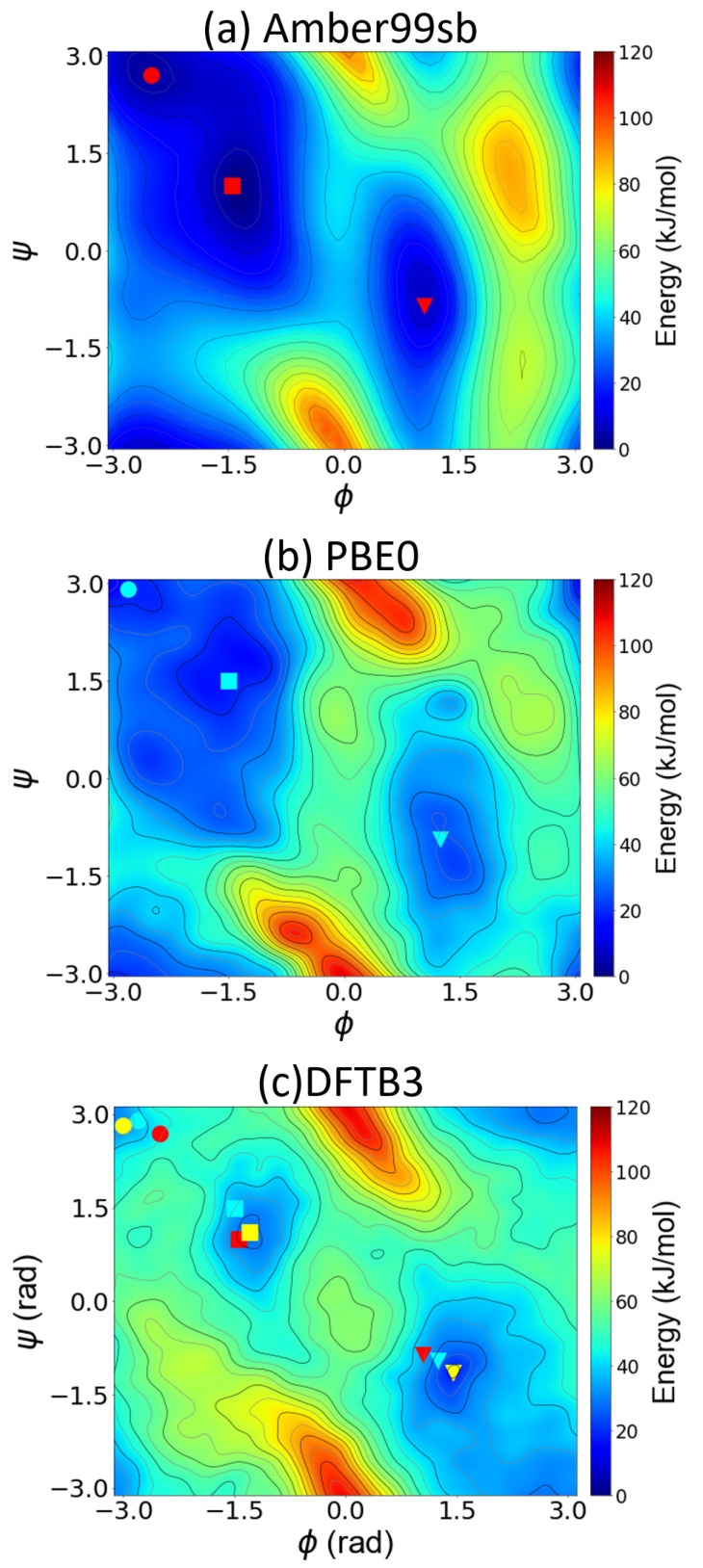
Two-dimensional potential energy surface of alanine dipeptide as a function of the backbone dihedral angles, ϕ and ψ, obtained from well-tempered metadynamics simulations using (**a**) classical MD from the Amber99sb force field, (**b**) DFT-PBE0 calculations, and (**c**) SCC-DFTB3 calculations. The red, cyan, and yellow points in panels (**a**–**c**) represent the local minima obtained using the Amber99sb force field, PBE0, and SCC-DFTB3, respectively. ●, ■, and ▼ denote the positions of the local minima.

(16) ΔA(ϕ,ψ)=ΔU(ϕ,ψ)−TΔS(ϕ,ψ),

where *A* is the free energy, *U* is the internal energy, *T* is the temperature, and *S* is the entropy. The internal energy contribution to the FES was calculated using the local average of the internal energy computed during the MD simulations on a (ϕ,ψ) grid. The entropic term, TΔS, was calculated from the difference between the internal and free energy (i.e., ΔA(ϕ,ψ)−ΔU(ϕ,ψ)). Figure 3 compares the potential energy surfaces obtained from the various methods. The classical force field predicts a higher internal energy than the DFT-PBE0 and DFTB3 methods in the region corresponding to ϕ=2 radians, which is also reflected in the FES in Figure 2. The differences between the DFT-PBE0 and DFTB3 FESs are mirrored here, as the barriers predicted by the PBE0 functional are higher than those calculated by DFTB3 in the region corresponding to ϕ=2 radians. The PES obtained from the classical MD, DFT-PBE0, and DFTB3 approaches differ most in the low-probability states (i.e., states with high energy values): DFTB3 and DFT-PBE0 predict similar locations of the local minima, whereas the classical Amber force field gives quite different results.

The entropic contributions to the free energy also exhibit significant differences (Figure S1). The classical force field predicts a low entropy compared with the DFT-PBE0 and DFTB3 approaches. The entropy maxima for all three cases are located near the same locations as their corresponding FES and PES maxima. The DFTB3 plot in Figure S1c shows that two of the minima correspond to C${}_{7\mathrm{eq}}$ and C${}_{7ax}$, while the third one located at (ϕ,ψ)=(−0.5,−3) radians does not correspond to a minimum in the FES or a well-defined structure. The classical force field severely underestimates the entropic contribution to the free energy because entropy is not explicitly included in the fitting of the force field. As such, our GPU-enhanced DFTB calculations support the recent claim [22] that the entropic contribution is *essential* for obtaining an accurate description of large peptides, especially for folding and unfolding processes. In particular, our DFTB3 calculations for the FES/PES qualitatively agree with computationally intensive DFT-PBE0 benchmarks, whereas classical force fields give significant errors.

Most importantly, the computational effort/time for our DFTB calculations is significantly less than that of full DFT (while still being more accurate than classical MD). As reported in a previous study by de Pablo et al. [22], the DFT-PBE0 calculations took 4.5 weeks to carry out a 1.5 ns metadynamics simulation. However, our DFTB metadynamics simulation on ADP (22 atoms) took only ≈18 h to obtain a 5 ns converged FES, indicating that our GPU-DFTB approach is nearly two orders of magnitude faster than DFT-PBE0. As noted previously, because the diagonalization algorithm scales as $O(n^{3})$ (where *n* is the matrix dimension), increasing the system size twice would incur an eight-fold increase in computational cost. Therefore, a system size of ≈80 atoms is well within the capabilities of our GPU-enhanced DFTB approach (i.e., a metadynamics simulation of 10 ns would take ≈21 days), which cannot be easily obtained with DFT-based metadynamics.

### 4.3. Large-Scale GPU-DFTB Metadynamics Simulations of Remdesivir

With our GPU-enhanced DFTB approach validated against the high-level DFT-PBE0 ADP benchmarks, we then proceeded with metadynamics calculations of remdesivir as a proof-of-concept example of a system that is impractical to calculate with full DFT. Remdesivir has garnered recent attention in treating various ailments [78,79,80] and is a structurally complex molecule consisting of three key fragments: an adenine analogue base, a pentose sugar unit, and a phosphoramidate side chain. Broadly, predicting the FES landscape of promising drug candidates can guide the calculation of binding affinities and/or transition pathways to accelerate the drug design process. As such, the use of accurate computational approaches to efficiently predict the FES (such as the GPU-enhanced DFTB approach used here) can provide essential thermodynamic information for directed structure-based drug design.

**Figure 4:**
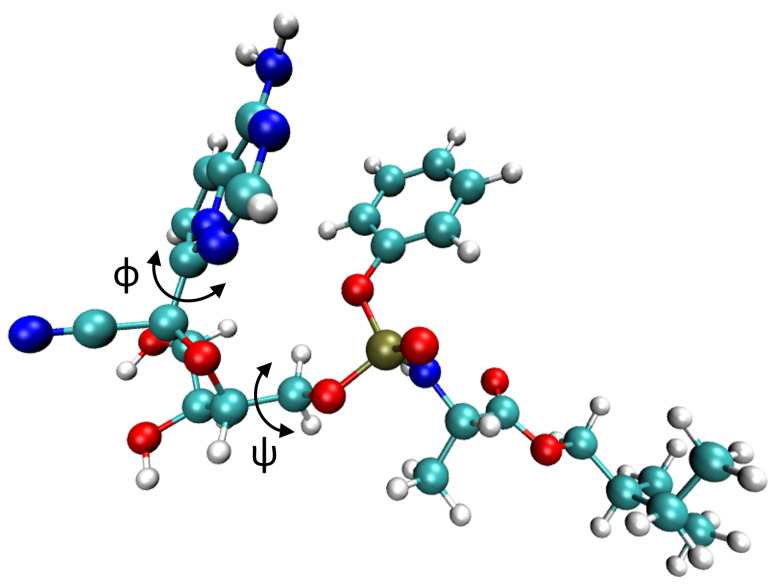
Molecular structure of remdesivir, which is composed of 77 atoms. The two dihedral angles, ϕ and ψ, are used to bias and analyze our calculations. The H, C, N, O, and P atoms are shown in white, cyan, blue, red, and yellow, respectively.

As mentioned previously, converged metadynamics calculations with full DFT on large chemical systems such as remdesivir are impractical. More specifically, previous DFT-PBE0 metadynamics calculations on the 22-atom ADP molecule required 4.5 continuous weeks of computing time [22], and simulations on the 77-atom remdesivir molecule at that same level of theory would take several months. As such, the remdesivir metadynamics calculations in this study are excellent extensions of our GPU-enhanced DFTB capability to highlight and test its computational limits. To compare our DFTB3 calculations against those of conventional MD approaches, we also carried out classical Amber force field metadynamics calculations. Figure 4 shows the structure of remdesivir with the dihedral angles, ϕ and ψ, used to bias the metadynamics calculations.

Before calculating the FES, we examined the convergence of our DFTB WT-MetaD simulations by calculating the free energy as a function of time. In general, when a metadynamics simulation is converged, the resulting FES profiles are similar within a constant offset. Figure S2, in the Supporting Information, depicts the FES calculated every 0.5 ns up to a total time of t=10.0 ns. We found that the FES did not change appreciably from t=9.0 to 10.0 ns (other than a constant offset), which indicates that our simulations fully converged. Figure S3 shows the one-dimensional free energies (extracted from the full-dimensional metadynamics calculations) along the dihedral angles ϕ and ψ as a function of simulation time. We found that the free-energy differences of −6.92 and −3.80 kJ/mol associated with the basins near ϕ=−2 and 2 radians and ψ=−2 and 1 radians, respectively, did not change appreciably, which indicated the FES calculations reasonably converged.

**Figure 5:**
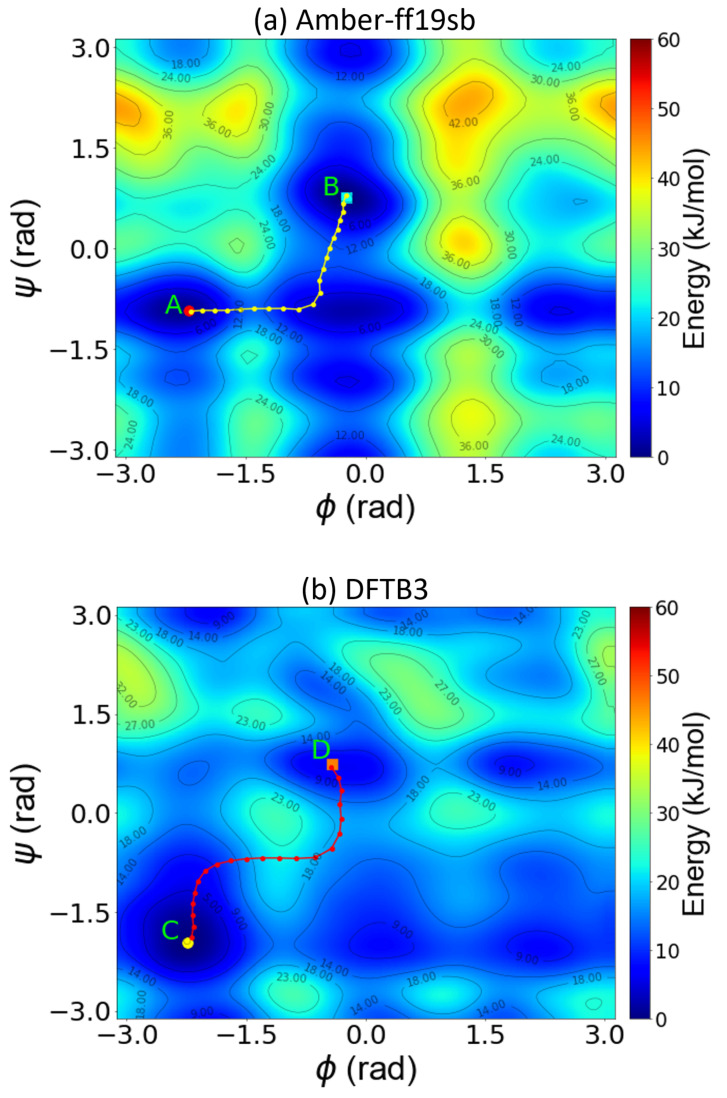
Two-dimensional free-energy surface of remdesivir as a function of the backbone dihedral angles, ϕ and ψ, obtained from well-tempered metadynamics simulations using (**a**) classical MD from the Amber-ff19SB force field and (**b**) SCC-DFTB3 calculations. Points A/B and C/D represent the dominant minima along the transition pathway (shown as a dotted line) obtained from the classical Amber force field and DFTB3 calculations, respectively.

Figure 5 depicts the FES of remdesivir projected onto the ϕ and ψ dihedral angles calculated from well-tempered metadynamics simulations at 300 K via the Amber-ff19SB force field (Figure 5a) and DFTB3 (Figure 5b) approaches. The energy barriers and transition pathways for each of the plots were computed using the nudged elastic band (NEB) method as implemented in the Metadynminer package [81]. Using these two CVs for remdesivir, we were able to identify two dominant minima in a Ramachandran plot (see points A/B and C/D for the Amber force field and DFTB3 approaches, respectively), which describe the metastable structures of the molecule.

There are clear differences between the DFTB3 and classical force field predictions for the FES. The most discernible difference is near the maximum at (ϕ,ψ)=(2.8,2.8) radians in the Ramachandran plot, which is less pronounced in the DFTB3 calculations. In addition, the Amber-ff19SB force field predicts a much larger barrier that spans the entirety of ψ at ϕ=1.4 radians, likely restricting conformational transitions across the dihedral angle. In addition, the DFTB3 calculations predict a much smaller barrier that spans the entirety of ψ at ϕ=2 radians, likely allowing conformational transitions across the dihedral angle. The local energy maximum predicted by DFTB3 near (ϕ,ψ)=(1.0,1.5) radians is surrounded by a valley that constitutes natural pathways between the two minima at (ϕ,ψ)=(−0.50,0.72) and (1.80,0.72) radians. The region near ψ=0.72 radians predicted by DFTB3 is significantly different as there are no prominent maxima. Both DFTB3 and Amber provide similar geometries for the dominant minima near (ϕ,ψ)=(−0.50,0.72) radians (points B and D in Figure 5). To assess the accuracy of the DFTB3 and Amber calculations, we carried out single-point hybrid-DFT calculations on remdesivir geometries extracted from points A, B, C, and D to understand which of the two approaches are consistent with the more-accurate DFT calculations. Table S1 in the Supporting Information shows that the hybrid DFT calculations predict the molecular geometry at point C to lie lower in energy than any of the geometries extracted from points A, B, or D. As such, this test indicated that DFTB3, which predicts point C to be the global minimum in Figure 5b, is consistent with full DFT (in contrast, the Amber calculations shown in Figure 5a incorrectly predict the global minimum to lie at point A).

**Figure 6:**
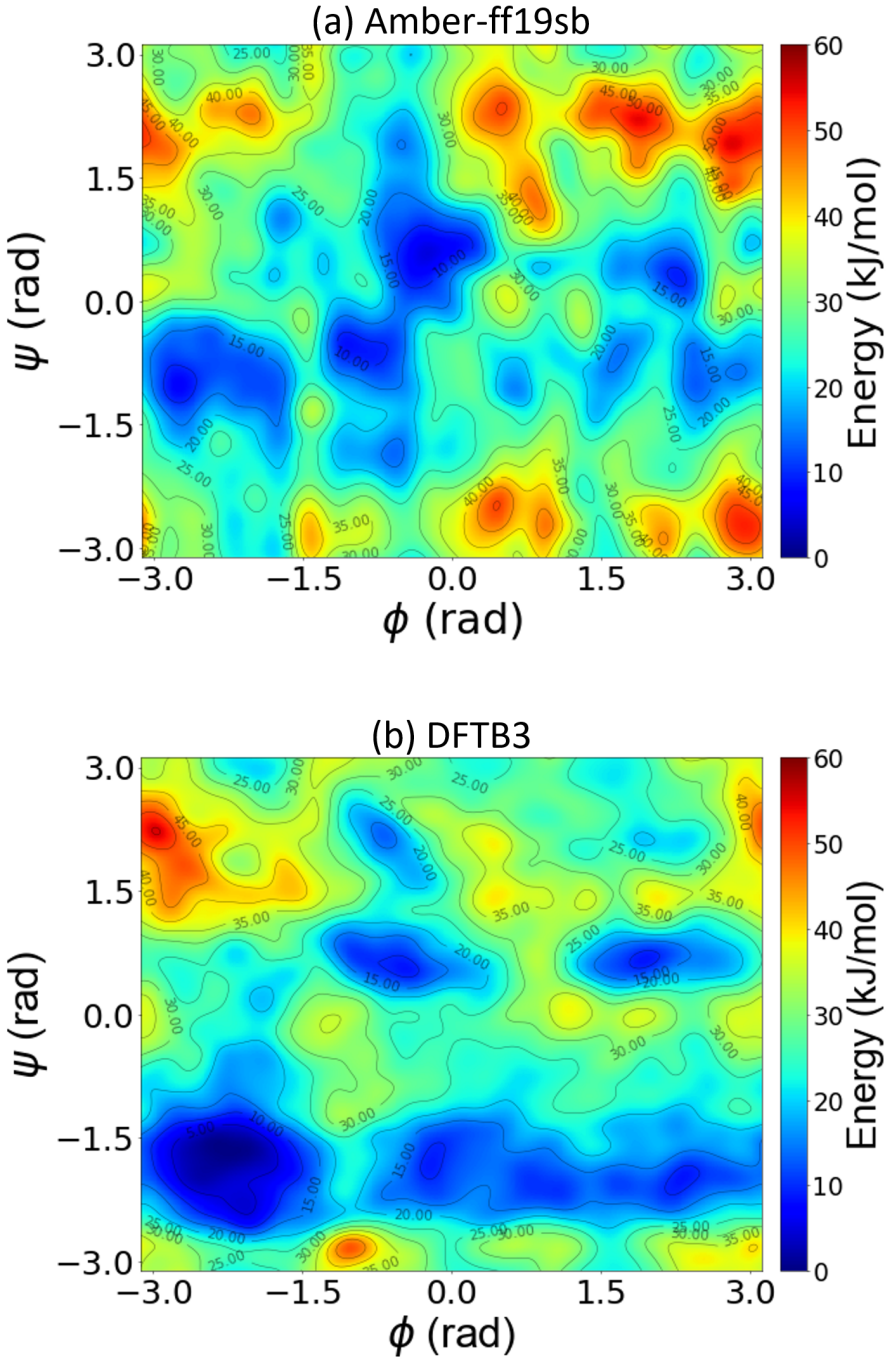
Two-dimensional potential energy surface of remdesivir as a function of the backbone dihedral angles, ϕ and ψ, obtained from well-tempered metadynamics simulations using (**a**) classical MD from the Amber99sb force field and (**b**) SCC-DFTB3 calculations.

Similar to our analysis on ADP, we investigated the origin of the FES differences by computing the contribution of internal energy and entropy for remdesivir. Figure 6 compares the potential energy surfaces obtained from the classical and DFTB3 approaches. The classical force field predicts a higher internal energy than the DFTB3 methods in the entire region near ψ=2 radians, which is also reflected in the FES in Figure 5. The differences between the Amber and DFTB3 FES are mirrored here, as the barriers predicted by the Amber calculations are higher than those predicted using DFTB3 in the region near ψ=2 radians. These differences are also observed for the global minimum, which the Amber force field predicts to be less stable. Similar to ADP, the remdesivir PES predicted by Amber and DFTB3 differ most in the low-probability states. Figure S4 shows the entropic energy surfaces obtained using various methods. The classical force field predicts a higher entropy than DFTB3 in the region corresponding to ϕ=2 radians, which is also reflected in the FES in Figure 5. The differences between the DFT-PBE0 and DFTB3 FES are mirrored here, as the barriers predicted by the PBE0 functional are higher than those calculated at the DFTB3 in the region near ϕ=2 radians.

In summary, our results emphasize the importance of including quantum effects for accurately probing the metadynamics of remdesivir. In particular, our DFTB3 and Amber classical field calculations give qualitatively different predictions for the remdesivir FES. To estimate the accuracy of these two computational approaches, we carried out benchmark tests showing that the DFTB3 results are much more consistent with full DFT than the Amber classical force field. The resulting errors in the Amber classical force field manifest themselves in the FES by overestimating the internal energy contribution, particularly in the high free-energy remdesivir configurations. Taken together, our results show that our GPU-enhanced DFTB approach is a promising approach for accurately calculating the long-term metadynamics of remdesivir, which cannot be easily carried out with full DFT calculations.

## 5. Conclusions

In conclusion, we have extended our GPU-enhanced DFTB approach to enable efficient simulations of long-term metadynamics calculations of complex biochemical systems. Carrying out metadynamics calculations on these large biochemical systems is a natural extension of our GPU-enhanced DFTB approach because the diagonalization of the Hamiltonian matrix is performed several times during a single MD trajectory, which can be accelerated with massively parallelized GPUs. To enable these large simulations, we also carried out these calculations on Microsoft’s Azure cloud platform to demonstrate the viability of cloud computing resources for quantum simulations.

After testing the performance of our GPU-DFTB approach on various hardware configurations for optimal performance, we verified the accuracy of our computational approach by calculating the free-energy surfaces of alanine dipeptide, which is a standard reference system for evaluating the performance/accuracy of enhanced sampling methods. In contrast to classical force fields, which give qualitatively different results than DFT-PBE0 benchmarks, we found that our GPU-enhanced DFTB calculations are in good agreement (with a much lower computational cost) with the computationally intensive DFT-PBE0 benchmarks. To further extend our GPU-enhanced DFTB approach, we also carried out a 10 ns metadynamics simulation of remdesivir, which is prohibitively out of reach for routine DFT-based metadynamics calculations. To the best of our knowledge, this is the first time that a quantum-based FES has been calculated for remdesivir for a relatively long sampling time of 10 ns. We found the free-energy surfaces obtained from classical and DFTB3 calculations differ signifcantly. Compared to DFTB3, the classical force field overestimates the internal energy contribution of high free-energy states in remdesivir, which produces dissimilar transition pathways that connect different minima on the free-energy surface. Taken together, our calculations and benchmark studies indicate that GPU-enhanced DFTB metadynamics is a promising technique for sampling the long-term thermodynamics of biochemical systems that require more accuracy than classical force fields but cannot be easily calculated with full DFT methods.

## Figures and Tables

**Table 1:** Comparison of timings for various hardware configurations for carrying out 8 SCC iterations on protease 6LU7.

Hardware Configurations		Wall Clock (min)
Number of CPUs	Number of GPUs	
40	4	23.43
20	4	7.74
10	4	7.98
8	4	7.89
4	4	5.59
8	2	5.87
4	2	5.17
**2**	**2**	**3.89**
8	1	6.05
4	1	3.95
2	1	3.93
1	1	32.45
8	0	14.74
1	0	59.09

## Data Availability

The MD trajectories obtained in this work and the in-house scripts can be downloaded from Github (https://github.com/Anshuman5/metadynamics, accessed on 16 January 2023).

## Supplementary Materials

The following supporting information can be downloaded at: https://www.mdpi.com/article/10.3390/molecules28031277/s1. Figure S1: Comparison of the entropic term, TΔS, of alanine dipeptide as a function of the backbone dihedral angles, ϕ and ψ, obtained from well-tempered metadynamics simulations using classical MD with the Amber99sb force field, DFTPBE0 calculations, and SCC-DFTB3 calculations; Figure S2: Convergence of the free-energy surface of remdesivir as a function of time with respect to the dihedral angles, ϕ and ψ, using well-tempered metadynamics; Figure S3: Free-energy difference between the basins near ϕ = −2 and 2 radians and ψ = −2 and 1 radians as a function of simulation time; Table S1: Relative energies of local minima (points A, B, C, and D in Figure 5 of the main text) calculated at the PBE0 and B3LYP levels of theory using the 6-311++g(d,p) basis set; Figure S4: Comparison of the entropic term, TΔS, of remdesivir as a function of the backbone dihedral angles, ϕ and ψ, obtained from well-tempered metadynamics simulations using classical MD from the Amber-ff19SB force field and SCC-DFTB3 calculations.

## Sample Availability

Samples of the compounds are available from the authors.
