# Supplementary material for: GPU-Enhanced DFTB Metadynamics for Efficiently Predicting Free Energies of Biochemical Systems
Source: Molecules. 2023 Jan 28;28(3):1277. doi: 10.3390/molecules28031277 (PMC9920250; doi:10.3390/molecules28031277)
Supplement: Supplementary file 1 [file molecules-28-01277-s001.zip › molecules-2156592-supplementary.pdf]

List of Figures

S1 Comparison of the entropic term, $T\,\Delta S$, of alanine dipeptide as a function of the backbone dihedral angles, ϕ and ψ, obtained from well-tempered metadynamics simulations using classical MD with the Amber99sb force field, DFT-PBE0 calculations, and SCC-DFTB3 calculations. 3

S2 Convergence of the free energy surface of remdesivir as a function of time with respect to the dihedral angles, ϕ and ψ, using well-tempered metadynamics. 4

S3 Free energy difference between the basins near ϕ = -2 and 2 radians and ψ = -2 and 1 radians as a function of simulation time. 5

S4 Comparison of the entropic term, $T\,\Delta S$, of remdesivir as a function of the backbone dihedral angles, ϕ and ψ, obtained from well-tempered metadynamics simulations using classical MD from the Amber-ff19SB force field and SCC-DFTB3 calculations. 7

List of Tables

TS1 Relative energies of local minima (points A, B, C, and D in Fig. 5 of the main text) calculated at the PBE0 and B3LYP levels of theory using the 6-311++g(d,p) basis set. 6

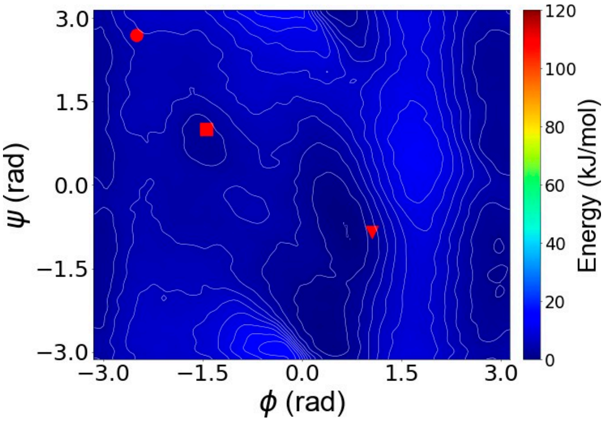

(a) Amber99sb

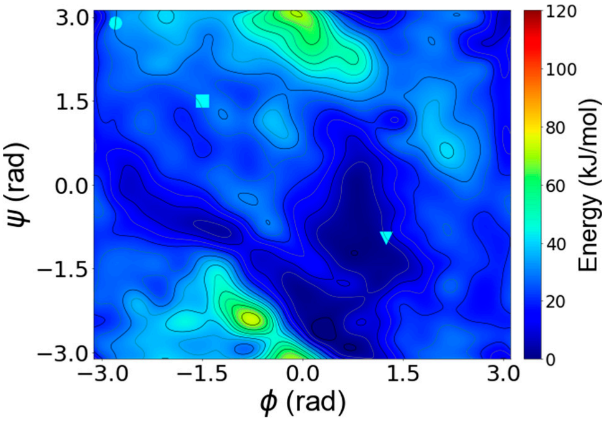

(b) PBE0

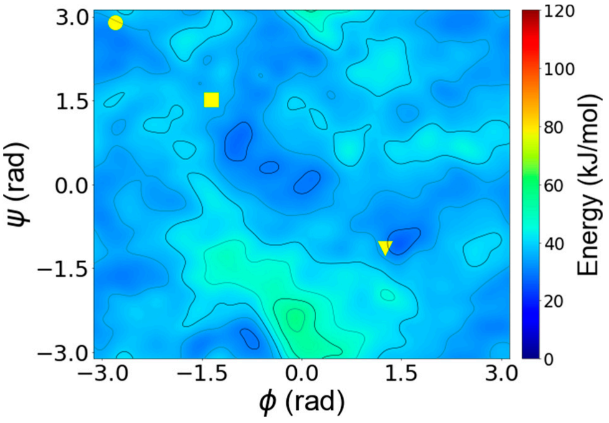

(c) DFTB3

Figure S1: Comparison of the entropic term, $T\Delta S$, of alanine dipeptide as a function of the backbone dihedral angles, ϕ and ψ, obtained from well-tempered metadynamics simulations using (a) classical MD with the Amber99sb force field, (b) DFT-PBE0 calculations, and (c) SCC-DFTB3 calculations. The red, cyan, and yellow points in panels (a), (b), and (c) represent the local minima on on the FES (cf. Fig. 2 in the main text) obtained using the Amber99sb force field, PBE0 and SCC-DFTB3, respectively. ●, ■, and ▼ denote the β, C_{7eq}, and C_{7ax} metastable structures, respectively.

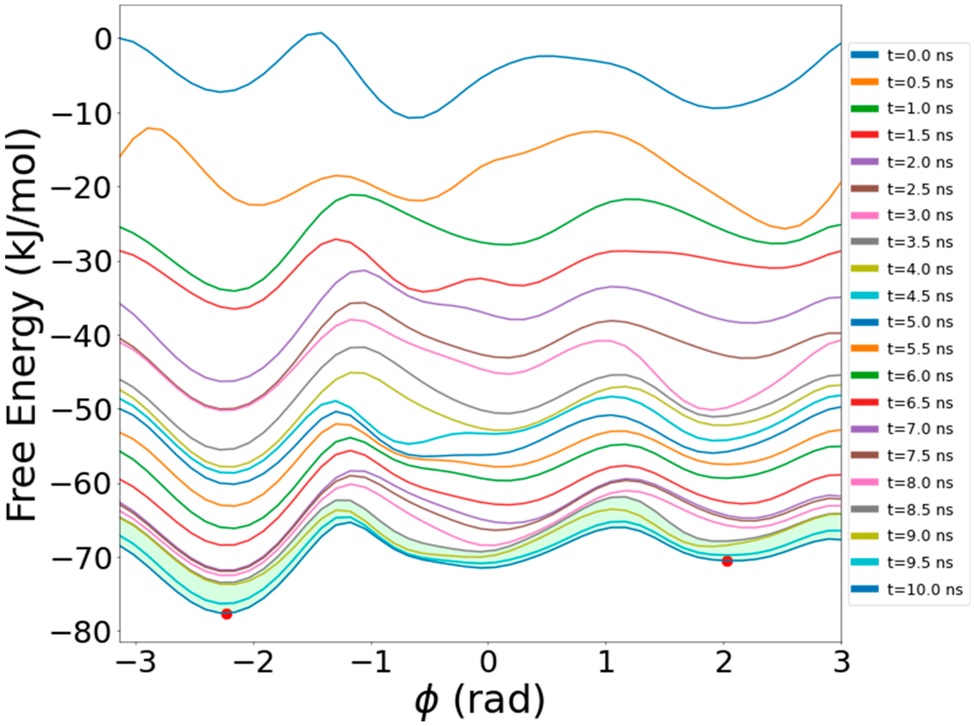

(a)

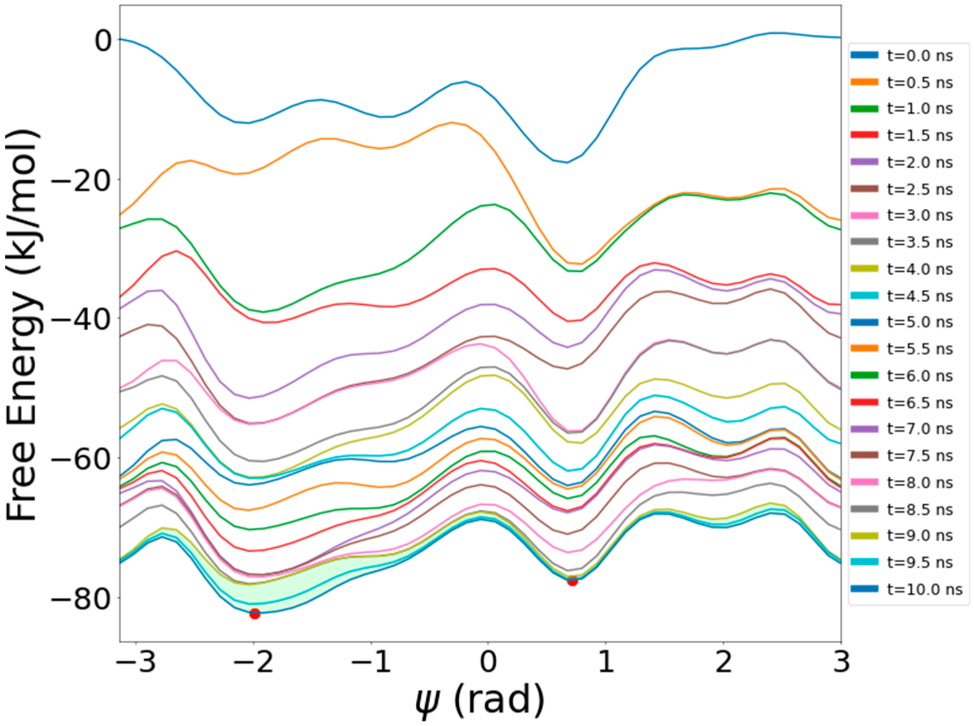

(b)

Figure S2: Convergence of the free energy surface of remdesivir as a function of time with respect to the dihedral angles, (a) ϕ and (b) ψ, using well-tempered metadynamics. The light-green shaded region in each plot indicates converged values. The red dots in panels (a) and (b) represent the local minima at which the free energy difference is calculated in Fig. S3.

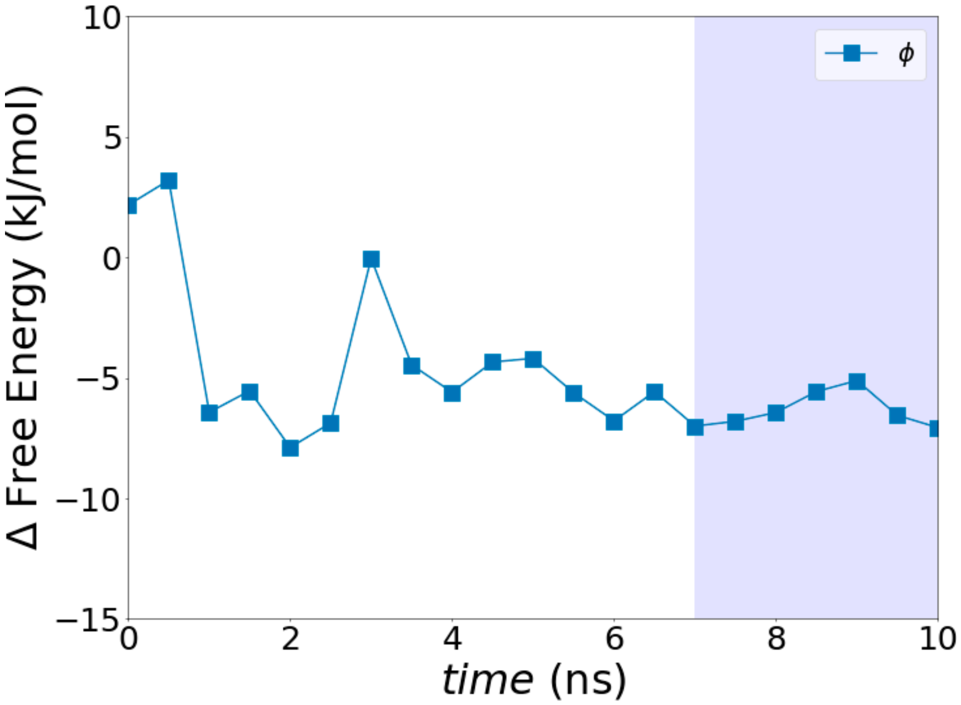

(a)

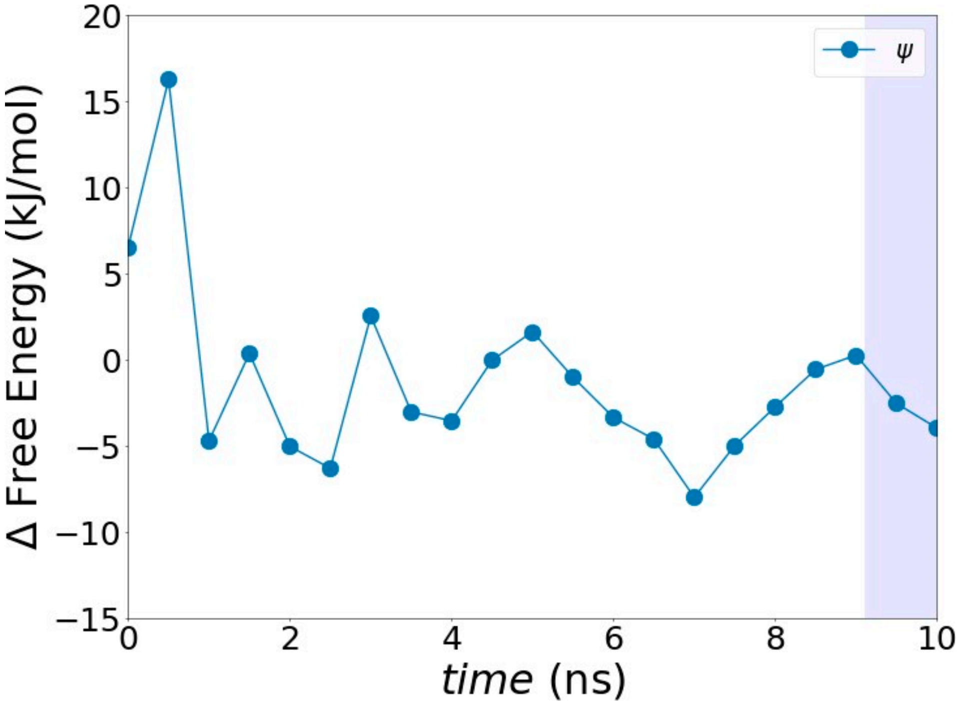

(b)

Figure S3: (a) Free energy difference between the basins near ϕ = -2 and 2 radians (shown as red dots at t = 10 ns in Fig. S2(a)) as a function of simulation time. (b) Free energy difference between the basins near ψ = -2 and 1 radians (shown as red dots at t = 10 ns in Fig. S2(b)) as a function of simulation time. The light-blue shaded region in each plot indicates converged values.

Table TS1: Relative energies of local minima (points A, B, C, and D in Fig. 5 of the main text) calculated at the PBE0 and B3LYP levels of theory using the 6-311++g(d,p) basis set.

	PBE0 (kJ/mol)	B3LYP (kJ/mol)
E_A	1.26	1.49
E_B	1.46	2.69
E_C	0	0
E_D	3.22	3.27

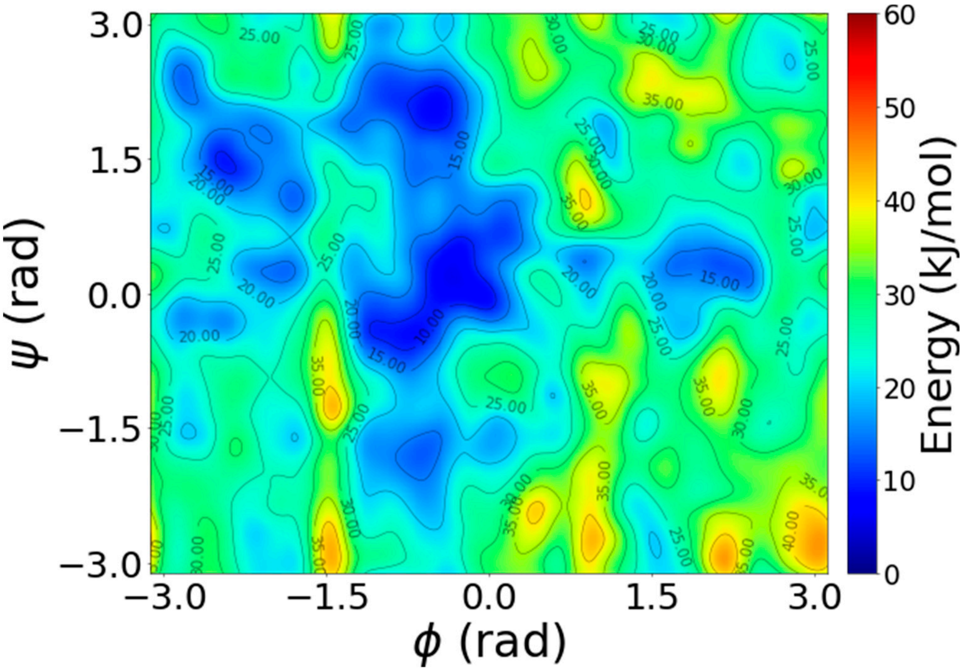

(a) Amber-ff19SB

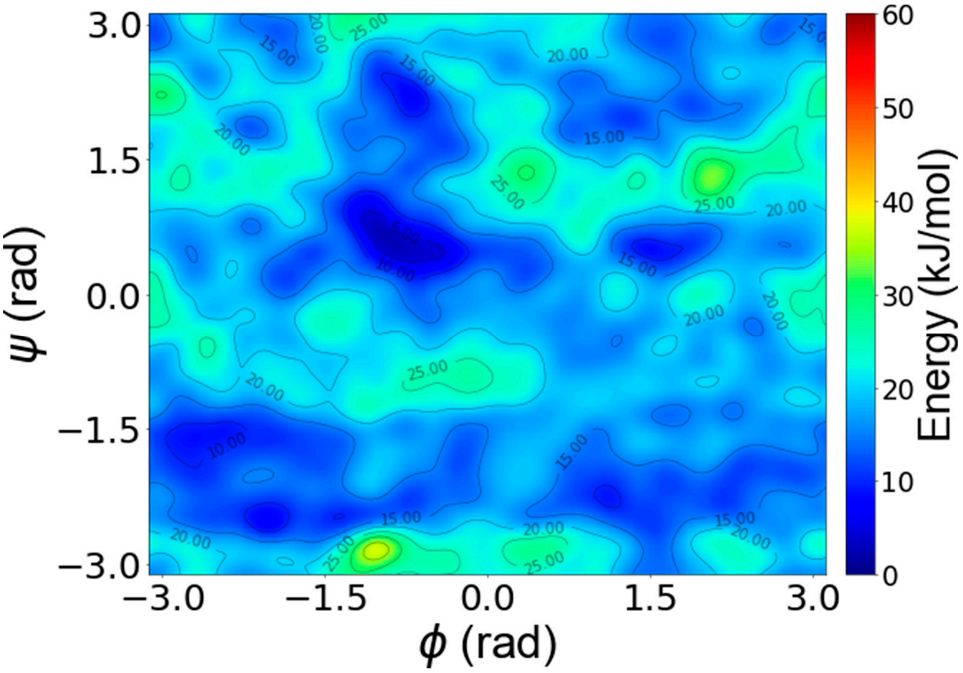

(b) DFTB3

Figure S4: Comparison of the entropic term, $T\Delta S$, of remdesivir as a function of the backbone dihedral angles, ϕ and ψ, obtained from well-tempered metadynamics simulations using (a) classical MD from the Amber-ff19SB force field and (b) SCC-DFTB3 calculations.
